# Exposure to job-related violence among young female sex workers in urban slums of Southwest Nigeria

**DOI:** 10.1186/s12889-022-13440-1

**Published:** 2022-05-21

**Authors:** Olutoyin Opeyemi Ikuteyijo, Akanni Ibukun Akinyemi, Sonja Merten

**Affiliations:** 1 Department of Epidemiology and Public Health, Swiss Tropical and Public Health Institute, University of Basel, Kreuzstrasse 2, Allschwil, 4123 Basel, Switzerland; 2 University of Basel, Basel, Switzerland; 3 Department of Demography and Social Statistics, Obafemi Awolowo University, Ile Ife, Nigeria

**Keywords:** Young female sex workers, Gender-based violence, Slums, Phenomenology

## Abstract

**Background:**

In Nigeria, many young girls are engaged in commercial sex work as a means of livelihood and support of dependent relatives. Although studies have documented some of the violence related issues among commercial sex workers, the plight of adolescent and young sex workers particularly in urban slums may be different in context and depth.

**Objective:**

This study explored the lived experiences of violence and health related harm among vulnerable young female sex workers in urban slums in Ibadan and Lagos, Southwest Nigeria. It also analyzed their coping strategies and survival mechanisms.

**Design:**

The study is cross-sectional and applied an interpretive phenomenological approach to this qualitative study through in-depth interviews.

**Participants:**

Young female sex workers ages (15–24 years) who reported having experienced violence were recruited for the study. Twelve participants completed the interviews out the 20 initially contacted.

**Data collection and analysis:**

Primary data were collected using in-depth interviews (IDIs). Data were transcribed using a phenomenological framework analysis. Participants’ reports based on life experiences were identified: lived experience “daily brothel life experience”; sources of violence such as law enforcement agents’ intermittent raids; violence experience with clients who often demanded sexual acts beyond the agreed scope; and coping strategies employed to mitigate the challenges.

**Settings:**

The study was conducted in brothels of two selected slum areas in Ibadan and Lagos, Southwest Nigeria.

**Results:**

The results showed that the major motivation for engaging in commercial sex work was for economic reasons. However, there are inherent risks involved particularly for the vulnerable young people. Stigmatization from the community, clients’ uncontrolled-aggressive behavior and harassment from law enforcement agents are some of the frequent violence experiences reported. Self-help coping strategies are usually employed to prevent or mitigate the challenges.

**Conclusion:**

The plight of this young people required policy and program attention towards alternative economic empowerment to rehabilitate those willing to leave the profession. Also the need to develop arm reduction interventions towards protection of young sex workers against violence.

**Supplementary information:**

The online version contains supplementary material available at 10.1186/s12889-022-13440-1.

## Introduction

Young female sex workers (YFSW) are often exposed to job-related, sexual, physical, economic and psychological abuse and violence perpetrated by clients [1–4]. The United Nations defines violence against women as “any act of gender-based violence that results in, or is likely to result in, physical, sexual, or mental harm or suffering to women, including threats of such acts, coercion or arbitrary deprivation of liberty, whether occurring in public or in private life” [5]. This often results in poor health outcomes in reproductive and mental health [6]. Achieving sustainable development goals (SDGs) i.e. SDG3, which focus on good health and well-being, and SGD5 on gender equality, are relevant to this study. Violence is being experienced among adolescents and young people forced in to sex work [7]. Gender-based violence (GBV) has remained a human rights violation [8], and is associated with negative health outcomes. Female sex workers (FSW) are found in various age groups, a fact rarely considered in the literature. The young age group 15–24 and adults 25–49 have however, different experiences. The age range considered is usually 15–49 years being the reproductive age, but experiences differ according to age groups in terms of health outcomes and other negative experiences [1, 3, 9]. Some of these studies have documented various human rights violations against sex workers (SW) by state actors i.e. police and other individuals. The focus on young women’s experiences with sex work over the years has received little attention [10].

Globally, FSW are among vulnerable groups of women as they experience violence and transmission of infectious diseases. The challenge of an acceptable definition of sex work ‘*consensual exchange of sexual intercourse between adults, for money or other goods, as a livelihood activity’* [11, 12] *remains a problem, and remains difficult to describe.* Cheryl describes it as the provision of sexual services for money or goods [13]. This study will adopt the definition of Cheryl, which is most appropriate in this context.

Job-related violence among YFSW is diverse in both developed and developing countries. In parts of sub-Saharan African countries, studies have demonstrated the prevalence of violence among FSW. A study in Kenya [14] found that 79% reported violence from a client or partner in the last 30 days. In Mombasa 87% had experienced GBV in their lifetime [15]. In Soweto, South Africa, GBV experience among FSW was attributed to both clients (46.8%) and police (18.5%) [16] and sometimes resulted in death [17]. In 5 Southern African countries, 70% reported experiencing physical and sexual violence in the past 12 months [18]. Violence was found to influence and increased the risk of acquiring HIV [19]. The WHO found that eliminating sexual violence against SW had the possibility of reducing 20% in new HIV infections [20]. Research showed a high number of SW with seropositive HIV status. One estimate from across Global South countries examined the number of SW and proportion with HIV showed that about 37% in Cameroon 38,582, 23% in Burundi, 51% Rwanda, 5% in Brazil, and 25% in Nigeria [19].

In Nigeria, sex work is not-illegal and the business continues to strive. However, most societal norms and values across Nigeria societies frown at it. In many instances, their rights are infringed and they are also been exploited and discriminated against. The Nigeria police and other security outfits often prey on the YFSW. In some cases, they are arrested and subjected to some inhuman treatment and exploitation. They are exposed to violence from clients as well as within the community.which makes police- perpetrated violence, client violence, and stigmatization thrive among SW. Nonetheless, Nigerian law does not categorically legalize nor criminalize prostitution. At night, FSW are present in the “red light” districts, hotels, bars and brothels.

YFSW who have experienced diverse forms of violence at the hands of clients, police and others have looked for ways of escape. A study from Nigeria showed how they devised coping mechanisms by setting boundaries, selecting clients and in some cases, they resorted to self-help and sometimes were armed with traditional medicine, charms and drugs as self-defense mechanisms [21], and oftentimes they preferred to relocate abroad or to other perceived peaceful areas in the country [22]. Some used self-restraint approaches [23], while others used comforting words to persuade and encourage themselves [24] to cope with violence abuse.

Previous studies have established that exposure to violence among female sex workers has a very high tendency for suicidal ideation, attempts of suicide, depression and post-traumatic stress disorder (PTSD) [17]. In addition to deleterious effects on mental health, violent experiences among YFSW have been linked to an increase in HIV infections and other related health problems. Studies have shown that YFSW exposure to violence further aggravates the risk of HIV infections [25, 26] and other genital diseases [15, 27–29]. YFSW were exposed to unprotected sex with their intimate partners, which increased the risk of HIV infection [30, 31].

Given the vulnerability of young people in slum areas to violence, and the gravity and consequences may be far greater for YFSW in slum areas. This study is therefore aimed at understanding the dimensions of job-related violence among this set of people. What is their experience and risk faced on their jobs? What safety nets do they have and what coping strategies do they adopt in coping with the risk of violence? These are the questions this study are aimed at unearthing. There is evidence from other geographies on female sex workers concerning their health, economics/poverty, and violence [29, 32–36], however, little research had used a phenomenological approach to focus on young women in sex work [10, 21]. While studies have reported on GBV and FSW, less attention had been given to the doubly vulnerable YFSW. There is a need to reduce exposure to violence, discrimination and extortion from police when addressing human rights violations among SW. We identified job-related exposure to violent experiences among YFSW, the health implications, the challenges faced during the course of their activities; and we identified their lived experiences and motivation to engage in sex work as well as their coping mechanisms.

## Methods

### Setting

The study was conducted in the major metropolitan cities of Lagos and Ibadan in Nigeria from January to June 2021. Lagos is the biggest metropolitan city with a population projected at 9 million, and Ibadan’s population was projected at 3,565,108 making it the third largest metropolitan city [37]. They are the major cities and have the largest slum areas where sex work strives on a daily basis. Although sex activities are primarily street prostitution, several brothels provided residential accommodations for SW as living space. These brothels are residences for SW where quick sexual services as short as 10 min are offered. The participants were YFSW in the Ekotedo area in Ibadan Northeast and Sango and Dopemu in the Agege Local Government Area (LGA), Lagos. These locations were also identified in previous studies as areas where sex worker activities thrived [9, 10].

### Study scope

The study scope is limited to young people involved in sex work because they were more vulnerable than adults. The study was also conducted in selected slum areas in South-west Nigeria characterized with poor infrastructure and more volatile to violence and some forms of social anomalies. The questions were linked to their most recent experience of violence.

### Study design

The study is a cross-sectional design that utilized interpretive phenomenological analysis (IPA) [38] to explore and interpret the lived experiences of job-related violence among young women engaged in sex work. This ethnographic study was an outgrowth of a larger project to explore various experiences of violence faced by young female adolescents in the slum areas and among young females who were already involved in sex work. IPA was used to explore and draw out individual lived experiences and to describe health challenges, motivation, violence and coping practices and consists of three key elements: phenomenology, hermeneutics, and idiography. The phenomenological component includes a detailed description of how the respondent’s world was formed. The hermeneutic aspect interprets and makes intelligible understanding and meaning out of the in-depth dual viewpoints. The idiography helps in understanding the uniqueness of individuals without any biases. These three components complement and help the researcher to pay attention to the participants’ detailed experiences [39]. IPA enables the researchers to describe participants’ experiences through their life stories [40]. This study used IPA to identify the meaning and interpretation of the participants’ experiences with violence as well as coping processes. The researcher maintained curiosity and interest in the participants’ daily life stories through open interviews, which helped shed more light on their lived experiences.

### Study population and sampling procedure

The population of the study included young female sex workers in the study locations. Initially, the method of convenience sampling enlisted 20 participants but only 12 agreed to complete the interviews. The selection was based on young females who had resided in the brothels for at least two consecutive months and who had experienced violence within the last three months. The interviews lasting approximately 30 min were audio taped in the open data kit (ODK) platform to guide against loss of interview information and were sent to the server immediately. The participants were duly informed about the purpose of the study and consent forms were signed although some declined signing because they wanted to protect their identities but gave verbal consent. Street-based commercial sex workers were excluded from this study.

### Study instruments

A semi-structured interview guide was utilized (see Table 1) with the help of experts from medical sociology, epidemiology and criminology. The participants were asked to share their daily-lived experiences in the brothel: 1) motivations for working in commercial sex work; 2) job-related health challenges; 3) exposure to violence; 4) adopted coping strategies; and 5) state actors and other individuals’ victimization experiences. Participants were asked to share their stories of different job-related violence since they had begun sex work. Two female research assistants working with local NGOs with Masters’ degrees in sociology and demography and experience in qualitative research conducted the interviews with the investigator.

**Table 1:** Guiding questions for the in-depth interview discussions

Theme	Issue	Guiding question
Lived experience		(1) How would you describe your activities in a typical day?
		(a) Probe: for most common activities involved in daily work
		(2) Could you tell me in what way you negotiate with your “emotional” partner or clients regarding protection (condom use)?
		(a) Probe: were there any risks considered while negotiating?
		(3) When were clients not ready to use a condom?
	Dynamics of their relationship with partners or clients and sexual violence	(4) What health problem do women like you face?
		(a) Probe: for rape, sex without a condom, painful and “rough” sex in the vagina and anus
		(b) Probe: for police arrest, drivers’ harassments, physical beating by clients
Coping strategy	Coping strategy to avert harm/violence	(5) How do you practice safe sex to avoid harm from your clients?
		(6) How do you mitigate arrest by police?
		(a) Probe: for negotiation, payment of unofficial fines, extending sexual favors
		(a) Probe: safety nets in case of challenges- financial, health, criminal, etc.?
Sexual gender-based violence (SGBV)		(7) Have you been assaulted or experienced stigmatization due to your job?
		(a) Probe: for other people known who have had similar experiences
		(8) Please describe your experiences of being assaulted or receiving negative or stigmatizing comments (narrate specific experiences)
		(a) Probe: What was your response? How did you handle these?
		(b) Probe: what type of services did you seek after the assault?
	Places and context in which violence occurs	(9) Could you tell me what usually causes disagreement or violence between you and your clients?
		(a) Probe: provide instances (narrative)
		(10). When you experience violence/harm from your clients, where does it usually happen?
		(a) Probe: in the hotel room, in the car, in the bar?
	Influence of societal pressure	(11) Could you tell me how people relate with you and describe your relationship with other people?
		(12) Are there times when people abuse you (verbally, discriminate or harm) because of work related identity?
		(10) Please could you share yours or others’ experiences of stigmatization with services like health care providers or the police?

### Data analysis

**Fig. 1:**
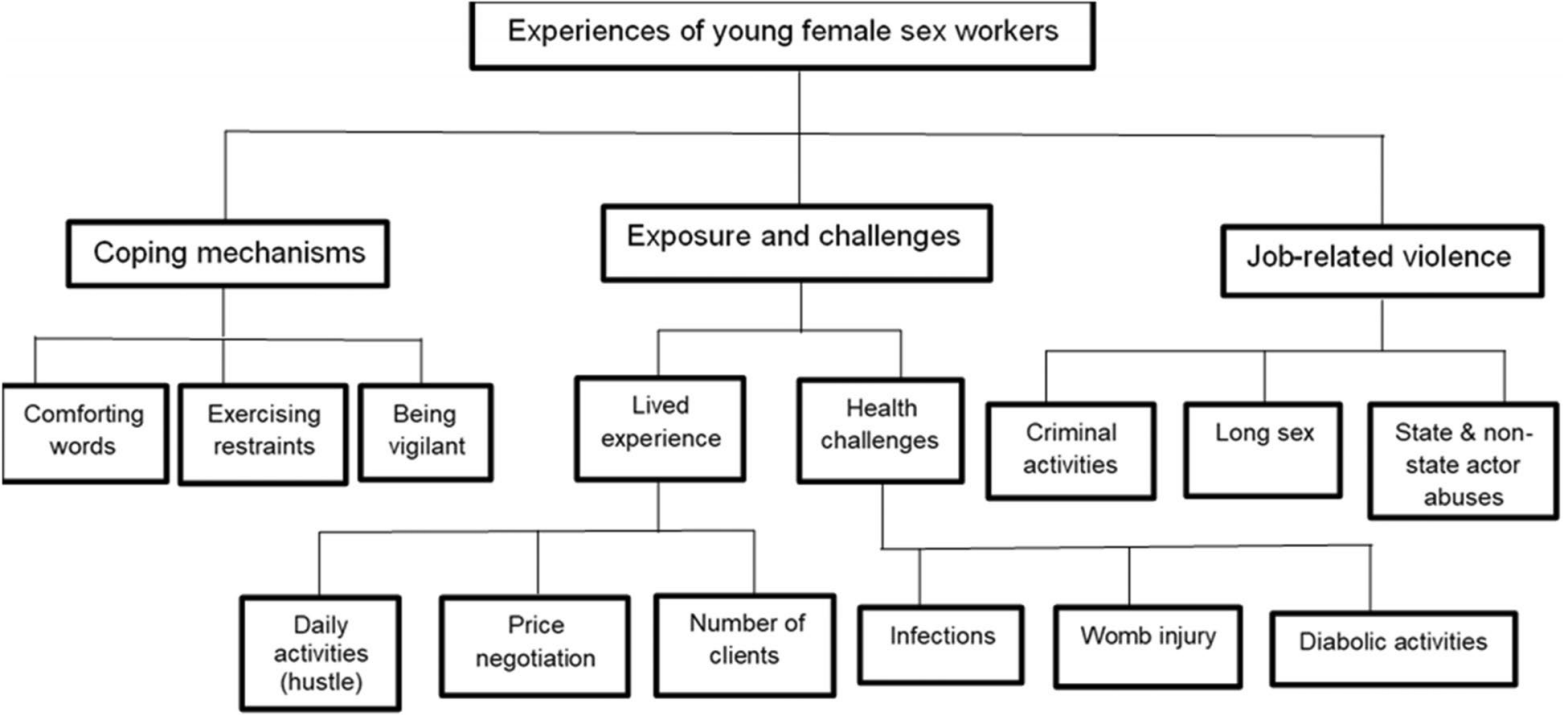
Coding tree for thematic analysis

The analysis of the data was completed according to research objectives. The interviews were collected using open data kits (ODK) and were transcribed verbatim. The Atlas ti version 8, a computer-assisted qualitative data analysis software (CAQDAS) was used. The in-depth reading of the transcripts and iterations, codes and themes were developed for inductive analysis. Framework analysis [41] was used to extract job-related violence experienced by YFSW. The steps recommended by Gale et al. were followed: 1) transcription; 2) familiarization with the interview; 3) coding; 4) developing a framework; 5) applying the framework; 6) charting data into a framework matrix; and 7) interpreting the data [41]. Reflexivity was guaranteed with additional field notes to guide the analysis and included observation, informal discussions with respondents and reflections after each interview. Reporting was guided by Consolidated Criteria for Reporting Qualitative Research (COREQ-32) [42]. A code tree was developed showing thematic analysis of the data (see Fig. 1).

### Findings

#### Socio-demographic characteristics of the respondents

The age range of the girls was 15–24 with a majority above age 20 (60%) while the rest were between ages 15–19. The minimum educational level attained was primary school while about half of the participants finished secondary school. Two of the respondents started in sex work less than 3 months before the survey, while the rest had been in the business for 2 or 3 years. All the participants claimed they solely engaged in vaginal sex with their clients. They all claimed using condoms during the intercourse; either female or male condoms were appropriate.

### Lived experience

The study findings revealed that young girls in the brothels were not residents of those communities where the brothels (i.e. drinking joints) were situated. A shelter was attached where the young women rented an apartment and brought their clients for daily sexual activities. They explained their activities, called “hustles” as they were involved with at least three or four men, and a maximum of 20 per day, with price differences.

*“…when I wake up, I will take my shower, after my shower, I will eat, relax, and maybe sleep for like some hours. If I do not feel like sleeping, I will go to the bar. After then during the evening I will dress up and hustle for customers.” (YFSW Ibadan).*

When the young women achieved their maximum number of clients on a daily basis, this was a high point for them.

*“On a normal day, good day, I can have up to 20 or 30 customers. Sometimes, I will not have up to ten, but I will get a lot of money. Okay let me say sometimes few people like 5 or 2 would enter my room, I would get a lot of money as well.” (YFSW Ibadan).*

They indicated their time limits for sexual activities with each client in order to achieve their daily target number and expected amount of money.

*“…Ehnn some time I get o, I just get customers, I can get like twenty in a day, and if I can cope I will accept them all. Highest is 30 min! Some spend more than 30 min, maybe at the end of the day; it’s mainly people that do stay long that spend that 30 min, some 5 min. It depends, all body systems are not the same.” (YFSW Lagos).*

### Motivation to engage in commercial sex work (CSW)

The YFSW commented that money was their motivation due to the high level of unemployment. Sex work was one of the available job options for young people and a means to meet their needs i.e. clothing, food and housing. YFSW regard this as a temporary job and something that they cannot tell family and friends they were doing.

*“…With this kind of place we are, you know what we do here now. We hustle to find money because is money that bring us here. Not that we are in our fathers house or our aunties’ house. Therefore, we came here to find money. So we don’t have choice (YFSW Ibadan).”*

### Exposure to violence

The violence experienced included physical, sexual, verbal and emotional abuses. Gender violence has been highly prevalent among women in Nigeria and Africa. YFSW experienced violence due to their age and suffered in the hands of clients and police officers. Some explained that clients would introduce diabolic activities or ideas during sexual intercourse; an example being that some clients would request to take away the discharge (semen) and offered extra money for such requests. It is important to define diabolic in the context of this study as “vile, cruel, worthy of an evil spirit, malicious, or immoral. Also, another definition is “having or showing a desire to cause someone pain for sheer enjoyment”. These two definitions are applicable to the experiences of young females in the sex work.

*“Yes sometimes customers usually fight us. When they pay for foreplay and enter to start asking for other things. In the course of collecting the money back, it can cause violence” (YFSW Lagos).*

Concerns were shared as to how the clients took advantage of them during sexual intercourse due to their age: forced sex, rough sex, amount of time, and condoms bursting during intercourse.

*“….when they go inside and we started, they will just change, that is it. For example, if they are not supposed to kiss, in the middle of the act, you just see them kissing you forcefully even if you push them; they force your face down”. (YFSW Lagos).*

*“..Some of them while having sex with you, will sometimes burst the condom and if that happens with me that is the end of the business because I cannot take another condom for the person and I will ask him to go. So this usually causes fight.” (YFSW Lagos).*

The participants explained that many clients were drunk or on drugs, which delayed their release during sexual intercourse. The challenge of defaulting the agreement was usually from the client’s side, which mostly resulted in violence.

*“Sometimes they will say, you did not allow them to “ejaculate”, as in release, because they have taken some stimulants or you didn’t allow them to touch your breast. One has hit me once and said ‘how can I be paying you and you did not allow me to touch your breast’ and that was not part of our agreement o. You know some customers are very stubborn, you will agree on something outside, they will demand for something else when you both are alone inside” (YFSW Lagos & Ibadan).*

Clients often demanded maximum sexual satisfaction, which invariably amounted to more time. If the request was declined, it often led to violence. At the intervention of the brothel manager, these issues were resolved without violence. Attainment of sexual satisfaction “ejaculation” mostly caused violence from clients and could only be resolved when clients were ready to pay a premium for more time to enjoy maximum satisfaction leading to sexual climax.

### Job-related challenges

Health related issues, which could be in the form of physical injury, vaginal infection, infertility, fear, and forms of diabolic incidents were problematic.

*“One day after intercourse, the client wanted to go away with the sperm in used condom but I objected. He told me he would give me more money, I insisted. This led to him being violent, but I insisted and he left without paying me.” (YFSW Ibadan).*

Long duration of sex from the clients raised many concerns and this had been a reoccurring challenge. Also, the challenge of non-payment for service among the clients takes young people for granted and is an abuse of human rights.

*“..The challenge is that some of these men will go outside and take drugs, and then they will come here and pay you for short time. Unfortunately, they will not be able to release on time, this will cause harassment, and insult and they want you to return their money.” (YFSW Ibadan).*

Commercial sex workers faced various risks but YFSW faced more due to their age. Some clients demanded to go “extra miles” with them like sucking their breasts. There were cases where other clients wanted them to participate as an accomplice in their criminal activities.

*“…some of us usually think any clients want to transfer sickness. Sometimes, they will come with charm and say they want to suck your breast or have sex without any protection (“skin to skin”). Then when you oppose, it will cause negative reaction.” (YFSW Ibadan).*

*“….there was a time, a guy came to hide cocaine in my room but I refused. If anything happens, it will tarnish my image, as my family do not know I engage in this type of job.” (YFSW Ibadan).*

### Abuses by state actors and other individuals

#### State actors’ abuses

Some participants suffered abuses from state actors like police officers. Police officers often demanded bribes. If there were no payments, the YFSW would be arrested and taken to the police station. Hence, most had devised a means of making money available for the police whenever the brothels were raided.

*“…. (Laughs), in this place like that, we face a lot of problems, police issues. Yes, about the police issue, if they come, you know this kind of job (laughs), it is free food for them.” (YFSW Lagos).*

*“…They do come here, but they do not arrest anyone because we pay them on a monthly basis. Each girl here contributes 3500 per month for police”. (YFSW Ibadan)*

#### Abuses from other individuals

YFSW experienced abuse from people around them. The name “Ashewo” is a household name for someone engaged in sex work. They were faced with clients’ harassments and in some occasions, clients’ partners coming to the brothel to confront them.

*“…they will say things like “ashewo” mean prostitute, don’t go and look for work to do, instead of just using yourselves to make money” and they don’t give us respect like other girls that are not in the street even the customers”. (YFSW Lagos).*

Clients made unpleasant gestures and when YFSW complained, they were adamant and never ready to apologize appropriately.

*“….the customers, if they do something you do not like and you tell them, if those that are responsible, they will understand and apologize, but some of them those ones that are not responsible, it will lead to para (fight) you understand. (YFSW Lagos).*

### Coping mechanisms

The use of comforting words, exercising restraints, and being ‘vigilant’ (i.e. they treated all clients as suspects who wanted more than sex) were devised as a means to cope with job- related violent encounters in order to survive.

“…If a client hires me till day break (overnight), I don’t usually sleep because of fear of whatever dangerous thing might happen” (YFSW Lagos).

“Yes they use condom but I will use my hand to put the condom and after which I don’t allow them to touch it again because of the fear of some that use “juju power” diabolic power and use girls for rituals”(YFSW Lagos/Ibadan).

YFSW were aware of health implications surrounding the sex work and in making conscious efforts not to affect their future health.

*“…I can’t allow them to do rough sex with me, and if they force me I will not open my leg very well I will just open it small. The reason is if you open leg too much it can affect our womb” (YFSW Lagos).*

Due to their youth and fear of reactions to their client’s violence, another coping strategy was remaining compliant in the form of “fear”. Some respondents declared they would do anything to satisfy a demand to avoid trouble or any violent act*.*

*“…If you bring customer inside, if you do not satisfy him well he will curse you, talk to you anyhow nonsense. If you curse him also, he will start fighting you. Therefore, as for me, I do not like that. If I take them inside, I will satisfy them well because as I am, I do not look for trouble. It is not because of trouble that bring me here.” (YFSW Ibadan).*

### Health awareness and challenges

A common health challenge was bleeding during sexual intercourse and this caused them need to recover from work.

*“…HIV, sexually transmitted disease, I have never been raped by a client before because he won’t even come in from outside if we don’t agree, but I have situations when clients are rough and caused me to bleed” (YFSW Lagos).*

Over the years YFSW have advanced in terms of knowledge of different infections and diseases the business exposed them to, nonetheless protecting themselves medically is low due to their vulnerable age. They usually patronized patent medicine vendors (PMV) for treatment in case of any traces of infections.

*“…When I need to receive treatment for anything, I go to private clinic as in (PMV), they are not rude to you for any reason because it is their work and you pay them for it. So, what I do is not their business”. (YFSW Lagos).*

The use of contraceptives was very important and all reported they used both female and male condoms during sexual intercourse. The only time they did not use contraceptives was when they had intercourse with their boyfriends; about half of the participants reported that their boyfriends were aware of their work.

*“I do use protection 100% and I use to tell them (customers)”. “….he will go out now; it's only my boyfriend that I can have sex with without protection”. (YFSW Lagos)*

Despite protection, few of the YFSW admitted they experienced “flesh to flesh” (unprotected) sexual intercourse with clients who were ready to pay a premium amount of money.

*“…okay, for me, sometimes if I see that you give me a lot of money, I have two things to do. Either I use a female condom or I use cotton wool. If you do not release inside me, then you will release on that cotton wool and I will remove it out. If you give me a lot of money, if I don’t have that female condom, that is what I do. But if I have, I normally use that female condom.” (YFSW Ibadan).*

With this agreement, they endeavored to protect themselves without the knowledge of their clients by using female condoms in such a way that was not known to the clients who demanded sex without protection.

Another health challenge was that of a diabolical act from the client.

*“Sometimes, they will bring charm for you that they want to suck your breast, or request for skin to skin ‘sex without condom’. Then when you refuse, the clients start acting negative.” (YFSW Ibadan).*

Injury during sex work activities was cited. Condoms burst during intercourse with clients, while others had experienced excessive bleeding which could make them need to recover for some days.

*“..Frequent penetration to the vaginal causes womb to shift from its original position. In addition, because we use bathroom and toilet together, one can contact infection.” (YFSW Lagos).*

## Discussion

Our findings indicated that female adolescents and young girls were willingly involved in sex work. Clients were the major perpetrators of violence which went beyond physical, sexual, and emotional to diabolical means. The YFSW experienced violence in the hands of clients, encountered economic frustration, and experienced abuses from both state actors and other individuals. This is similar to experiences of their counterparts in Ghana [43]. The narratives of lived experience of YFSW on a daily basis in the brothels [29] relied on the hope of getting paid by customers to cover their daily needs. The rate of customer flow depended on the profit for the day, and mostly the sexual activities were a short-term experience to allow for more clients. Commissions were paid to the group leader “chair lady” and the brothel owner on weekly basis. The major motivation expressed by YFSW was receiving money on a daily basis, which kept them in the business.

In consonance with previous studies [1–3, 44, 45], clients were one of the major perpetrators of violence among FSW and YFSW suffered more. Due to their age, greater exploitations were encountered: forced sex, free sex, unprotected sex, physical violence, longer time beyond the bargained time for sexual satisfaction, and diabolic requests. However, this study corroborates previous studies by showing that YFSW are at greater risk of job-related violence, sexually transmitted infections, HIV and other reproductive health outcomes [43, 46, 47].

Health challenges were one of the major concerns as implications of violence. The YFSW understood the health implications of their business and made conscious efforts of health protection by using condoms. Although a majority agreed that they used condoms for all customers, very few stated that they occasionally had sex with clients without condoms, which corroborates with previous studies [31, 48]. Excessive bleeding, genital infections, sexually transmitted diseases, and shifting of the womb, which could potentially cause infertility in the future, were concerns being consistent with previous studies [15, 27, 28].

YFSW suffered abuses in the hands of state (police and health workers) and non-state (clients and other people) actors. Police arrested them for no apparent reason. Demand for money as an entitlement was a major concern, which was in conjunction with previous studies [34, 49]. However, YFSW had devised several means of coping: being vigilant, setting boundaries, self-restraints, expressing comforting words to the clients and relocating from a particular location [21–24]. These coping mechanisms are in tandem with findings from previous research. The participants demonstrated coping by satisfying the clients’ sexual urges to avoid any violent attacks. Others tried to cope by being vigilant of any clients that approached them for unusual sexual activities.

There are issues of policy and program recommendations emanating from the findings of this study. There is a need for enlightenment and social orientation by government and non-governmental agencies, making violence against YFSW a public health and human rights priority on local and national policy agendas. The agenda should include work environment conditions, gender and economic inequities, stigma and security of lives. Interventions like “Naija-girls” in Lagos state targeting adolescents and young peoples’ sexual and reproductive health should focus on YFSW, as there is potential to influence their decisions. Health care providers must be more sensitive to young SW and create enabling environments. There is also the need for health workers to target YFSW for health interventions because of their naivety due to their relative lack of power because of both their gender, and their status as young females. Sensitization of the police and anti-violence activities among clients as well as a broader discussion on how to better protect young women by the state should be prioritized. Female police should check on brothels and ensure the welfare and health safety of this group. Monitoring and evaluation of this program will make a structural contribution and provide guidance on how best to meet the demands of highly vulnerable young females in sex work. Findings from previous studies [50, 51] from Nigeria focused on YFSW in relation to HIV while less focused and ignored young females sex workers lived experiences in the brothels, which will invariably increase their vulnerability. The study therefore encourages that more research be conducted on violence experienced among adolescents and young females who are engaged in sex work activities and on their health implications, future aspirations, and their fears.

### Strengths and limitations

One key strength of the study was that the data collected were strictly from young females in the commercial sex work industry who resided in the slums. Additionally, they were residents in brothels and worked full time in sex work with varying experiences.

This study had some limitations. The number of participants were few due to the age range considered and they were not too confident in sharing their experiences despite the assurance of anonymity and confidentiality. Second, since the present study consisted of self-reporting interviews, questions on personal healthcare received passive responses from almost all due to nature of their jobs. Getting them to respond to the interviews took time, until we assured them that we would move quickly with our discussion. Fourth, most of the respondents were migrants; they moved to locations where there were no families nor relations to recognize or apprehend them. Nevertheless, the interviews have provided us with vital information for this study.

This study made it clear that YFSW suffer much violence in the hands of clients and police as well as stigmatization in the community. Young female sex workers must be part of inclusive interventions, as this group will later in life transit into the society by either marriage or childbirth. Law enforcement agencies should relate responsibly with this group paying attention to their fundamental human rights. Therefore, working with law enforcement officials and health care providers may be the most promising next steps. It should also be made a human rights and public health priority.

## Conclusion

The study showed that exposure to violence further exacerbates the plight of younger girls who are involved in commercial sex. Rather than exploiting and criminalizing this vulnerable group, there is need for proper regulation and protection for them.

## Supplementary information

**Additional file 1:** Exposure to job-related violence among young female sex workers in urban slums of Southwest Nigeria.

## Data Availability

The dataset presented in this article are not publicly available, because it contains information that could compromise the privacy of the interviewees and a breach of agreement. Request to access the dataset can be directed to the corresponding author.
